# Spiral nematodes, soil microbiome and micronutrients increase chickpea drought susceptibility but do not induce symptoms of the emerging health issue

**DOI:** 10.1038/s41598-025-23475-0

**Published:** 2025-11-13

**Authors:** Victoria A. Marchesini, Jennifer Town, Mario Tenuta, Fernanda Gouvea Pereira, Lana Shaw, Shaun Sharpe, Jeff Schoenau, Michelle Hubbard

**Affiliations:** 1 Swift Current Research and Development Centre, Agriculture and Agri-Food Canada, Swift Current Saskatchewan, Saskatchewan, Canada; 2 Agriculture and Agri-Food Canada, Saskatoon Research and Development Centre, Saskatoon, SK Canada; 3 Faculty of Agricultural and Food Sciences, University of Manitoba, Winnipeg, MB Canada; 4 South East Research Farm, Redvers, SK Canada; 5 Department of Soil Science, University of Saskatchewan, Saskatoon, SK Canada

**Keywords:** Chickpea, Emerging health issue, Drought, Nematodes, Soil nutrients, Microbiome, Plant sciences, Plant physiology

## Abstract

In 2019, an emerging health issue was noted in chickpea in Saskatchewan, Canada. Symptoms included apical wilting, branch chlorosis and necrosis. The causes remain unclear. In 2023 these symptoms appeared on one side (“unhealthy”, UH), but not the other (“healthy”, H), of a dry field in Redvers, Saskatchewan. To test the hypothesis that *Helicotylenchus*, or spiral nematodes, and differences in soil microbiome and nutrients, in combination with drought, contribute to these symptoms, chickpea were grown in H and UH soil, and well-watered, or exposed to drought. Plant height, number of nodes and pods, chlorophyll fluorescence (Fv/Fm), biomass, foliar and root-rot symptoms, soil nutrients, nematodes and soil microbiome were assessed. Symptoms more consistent with drought than the emerging health issue developed. When chickpea was exposed to drought, symptoms were more severe in UH soil. Height and Fv/Fm were lower in UH soils. Foliar symptoms were more severe and spiral nematodes more abundant in UH soils. All parameters were affected by drought. Concentrations of K^+^ and Mg^+^ were higher in H soil; Ca^+^ concentration was higher in UH soil. Microbiome community composition, including bacteria, fungi and oomycetes, differed between H and UH soils, however, no pathogens that could be responsible for symptoms were more abundant in UH soils. Nematodes and other soil factors increase the impacts of drought but are not sufficient to induce the emerging chickpea health issue in pots. In the field, the root growth restrictions due to nematode feeding may lead to chickpea emerging health symptoms.

## Introduction

Chickpea (*Cicer arietinum* L.) is an important source of dietary protein for large populations around the planet, including India, Pakistan and Turkey^1^. Currently the demand for chickpea surpasses its production. Consumption has expanded rapidly due the potential benefits of chickpea to human health^2^, its potential to replace animal protein^3^ and its role in nitrogen fixation in the rotation with non-legumes, such as cereals and oilseeds^4^. In North America, chickpeas are grown mainly in the northwest of US and southwest Saskatchewan, Canada. In Saskatchewan, acres planted to chickpea have varied greatly over the last few decades (*Crop Insurance corp. SK*,* 2023*). These fluctuations have been driven by the many risks to chickpea production, especially ascochyta blight, caused by fungal pathogen *Ascochyta rabiei* (Pass.) Labr. ^5,6^

Biotic factors other than ascochyta blight, and abiotic factors, can also threaten chickpea production. In 2019, an emerging plant health issue was observed in chickpea in southwestern Saskatchewan, Canada. This health issue is characterized by discoloration of leaf tips, chlorosis, wilting or death of upper portions of plants and sometimes, plant death^7^. The issue can be localized in patches or spread across the entire fields. The symptoms are not present at the seedling stage. They generally begin following rainfalls and during chickpea flowering or early podding, often less than two months after seeding. The occurrence and severity of this health issue have varied since 2019, both spatially across the province and from year to year. Drought is suspected to contribute to this issue^8^.

Chickpeas are usually planted under good soil moisture, but for pod production and maturity, a certain degree of drought stress is needed at the end of the season. Although adapted to dry climates, excessive water stress or terminal drought constrain primary productivity in chickpeas, limiting root growth and reducing yield^9^. Droughts are common in the chickpea-growing regions of the Canadian prairies, leading to stress for chickpea, which, combined with other factors, may make this crop more at risk of the emerging health issue.

Recent studies have identified growing concerns about nematode infestations in pulses and soybeans across the Canadian Prairies^10^. A survey of 93 pulse fields in this area found infestations of pin (*Paratylenchus*) and spiral (*Helicotylenchus*) nematodes, among others^8^. Nematodes such as *Helicotylenchus* and *Paratylenchus* compromise the plant’s ability to absorb water and nutrients by impairing the root system and were detected in a survey for the chickpea health emerging issue in Saskatchewan^8^. Nematodes also contribute to the development of disease by puncturing root epidermal cells, which facilitates the entry of other pathogens into plant tissues^11^.

The microbiome of chickpea can be influenced by multiple biotic and abiotic factors including soil moisture, preceding crop and cultivar selection^12^. In particular, drought can exert strong selective pressure on chickpea microbiome composition, reducing microbiome diversity, and the influence of chickpea variety on microbiome^13^. Multiple specific bacteria and fungi have been associated with either chickpea health or disease states, underscoring the importance of the microbiome in shaping plant health^14^.

**Fig. 1:**
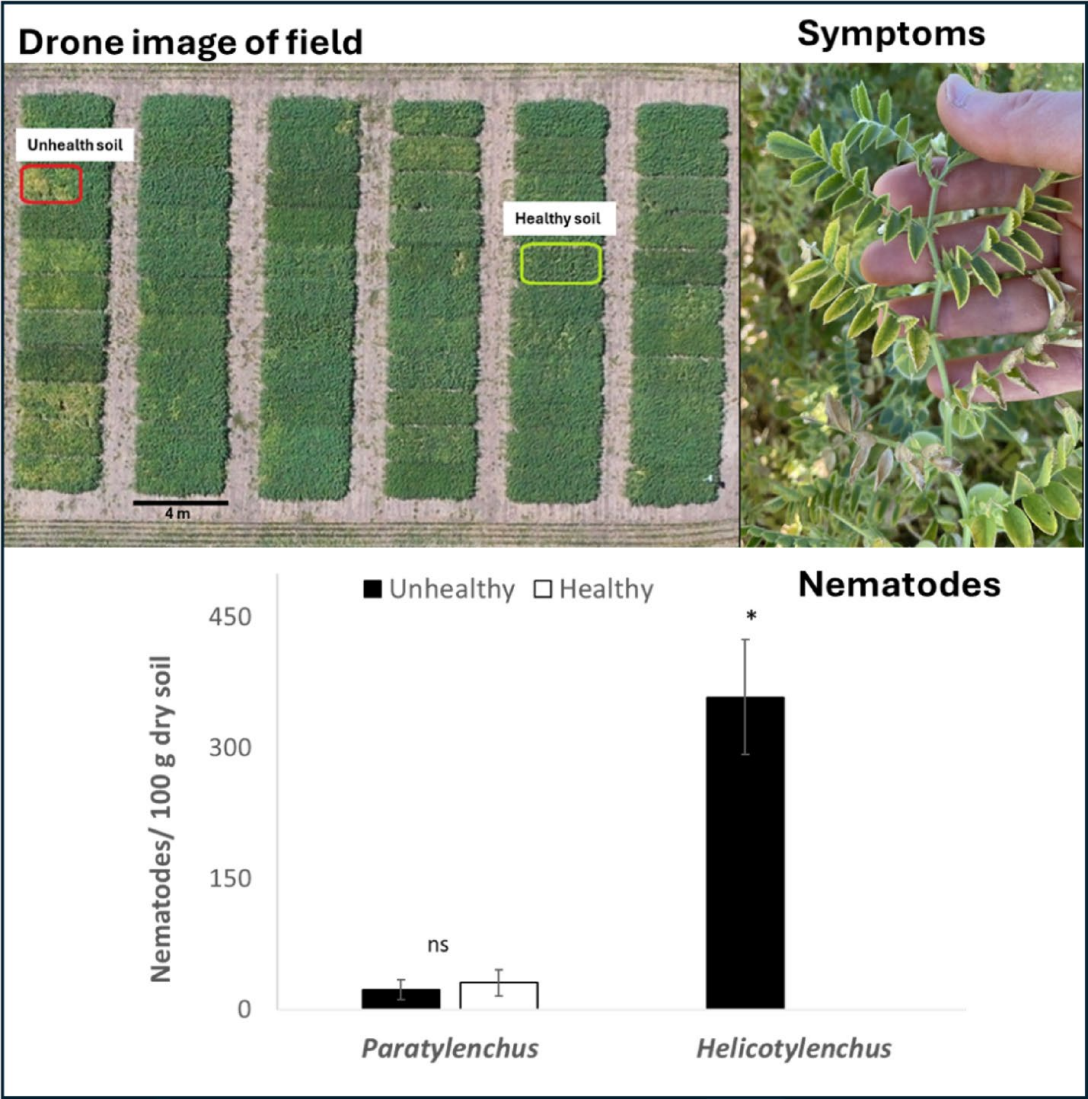
A drone image of field site showing the healthy and unhealthy plots (photo credit, R. Moroz) and photo symptoms of the chickpea emerging health issue showing chlorosis of leaf edges, near the top of a plant, from Redvers, Saskatchewan (photo credit, M. Hubbard). C) Number of pin (*Paratylenchus*) and spiral nematodes (*Helicotylenchus*) per 100 g of dry soil for healthy (H) and unhealthy (UH) soil. Asterisk (*) means statistically and ‘ns’ means not statistically different respectively (Kruskal-Wallis, p < 0.05).

This project aimed to test the hypothesis that the symptoms of the chickpea emerging health issue would be induced by the combination of spiral nematodes and drought. We planted chickpea in field soils in which the emerging health issue had developed (termed unhealthy, UH), and in field soils in which these symptoms had not appeared (termed healthy, H). The field from which the H and UH soils were collected, and symptoms, are shown in Fig. 1. We exposed chickpea in both H and UH soils to well-watered, moderate or severe drought in the greenhouse.

## Results

### Soil parameters

Soil nutrients were significantly different between H and UH soil (*p* < 0.05). The extractable K^+^ and SO_4_^−^ were almost double in H soil while Ca^+^_,_ NO_3_^−^ and PO_4_^−^ were higher in the UH soil (Table 1; Supplementary Table 1 for ANOVA tables). Other nutrients such as Mg^+^, Mn^+^ and Na^+^ were also higher in H soil (*p* < 0.05). Electrical conductivity (salinity) was significantly higher in H soils (*p* < 0.05). However, the difference between 1.1 and 0.96 mS/cm is unlikely to have biological importance as both values are considered non-saline. Total carbon and pH were similar between the two types of soil (*p* = 0.72). Organic carbon was slightly higher in H soil (*p* < 0.05). There was a small, significant difference in texture between soil type. The UH soil had slightly higher sand and lower silt content than the H one (*p* < 0.05). Textural variations across short distances (meters) in soils on the prairies arise from variable sand, silt and clay contents of glacial parent material derived from meltwater as well as redistribution of clay in erosion events.

**Table 1:** Soil extractable nutrients, total and organic carbon, pH, electrical conductivity and texture of healthy (H) and unhealthy (UH) soil used. Asterisk (*) indicate differences between H and UH for the parameter in question (*p* < 0.05).

	Healthy (H)	Unhealthy (UH)
K^+^ (mg/kg)	339.0	174*
SO_4_^−^ (mg/kg)	11.5	6.8*
NO_3_^−^ (mg/kg)	28.6	33.2*
PO_4_^−^ (mg/kg)	15.1	21.1*
Total-C (mg/kg)	3.0	3.0
Org-C (mg/kg)	2.6	2.0*
Cu+ (mg/kg)	2.0	3.4
Fe+ (mg/kg)	18.0	15.2
Mn+ (mg/kg)	28.2	20.4*
Zn+ (mg/kg)	2.7	1.9
Ca+ (mg/kg)	4633.3	6643.6*
Mg+ (mg/kg)	779.2	485.0*
Na+ (mg/kg)	24.0	18.4*
pH	7.6	7.7
EC (mS/cm)	1.1	0.96*
Sand (%)	54.1	57.9*
Clay (%)	22.8	22.9
Silt (%)	23.0	19.1*

### Soil biota: nematodes

In the UH soil, collected from the field shown in Fig. 1, in which symptoms of the chickpea emerging health issue developed (Fig. 1), a significantly higher number (Kruskal-Wallis, *p* < 0.05) of spiral nematodes (*Helicotylenchus spp*.) were found. The second most abundant group of nematodes, the pin nematodes (*Paratylenchus spp.*) did not differ between soil condition (*p* = 0.37).

### Soil biota: bacterial, fungal and oomycete community composition

**Fig. 2:**
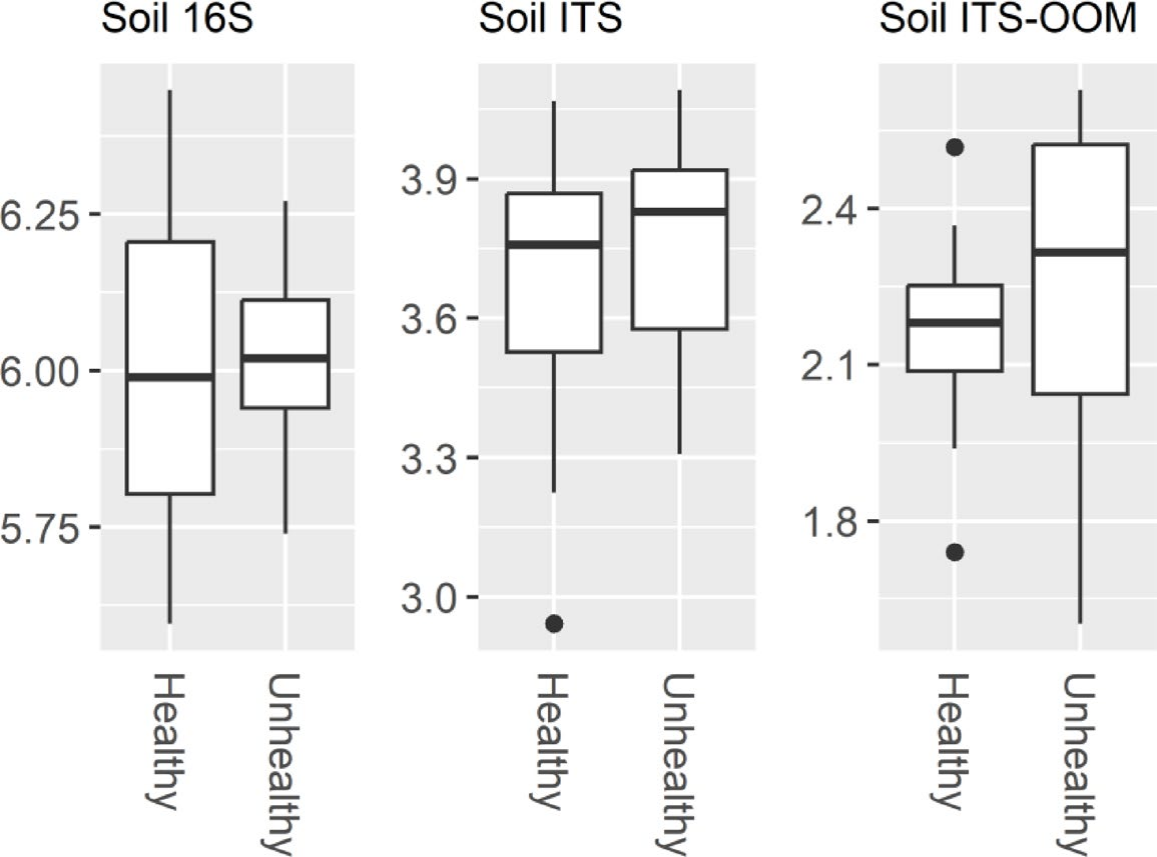
Shannon H’ diversity for 16 S, internal transcribed spacer (ITS) and ITS-oomycete (ITS-OOM) libraries from Healthy and Unhealthy soil samples. All libraries were rarefied to the same number of reads prior to alpha-diversity calculations, 5000 reads/sample for 16 S and ITS, and 1000 reads/sample for ITS-OOM. There were no significant differences between Shannon values for H and UH soil in either the bacterial (16 S) fungal (ITS) or oomycete (ITS-) communities (Kruskal-Wallis, *p* > 0.05).

**Fig. 3:**
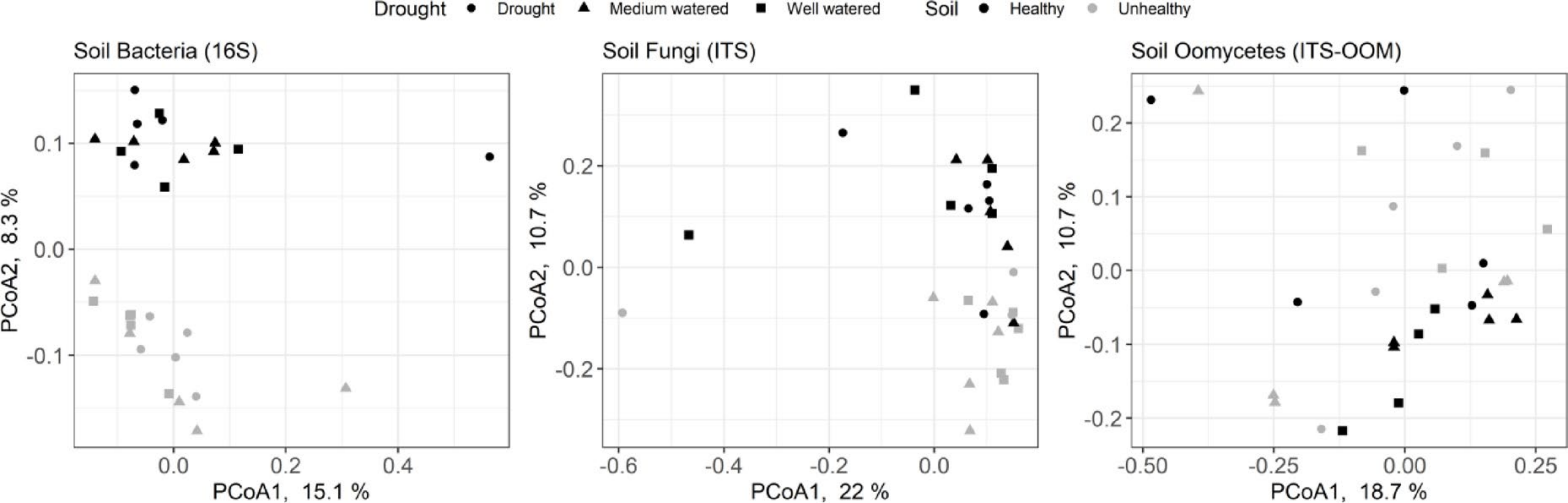
Principal coordinate analysis of Bray-Curtis dissimilarity to visualize between-group differences for bacterial (16 S), fungal (internal transcribed spacer, ITS) and oomycete (ITS-OOM) soil communities.

Alpha diversity, as measured by the Shannon H’ of the bacterial, fungal and oomycete soil communities, was not significantly impacted by soil, drought or the interaction between soil and drought (Fig. 2, Supplementary Table 2). The same was true of fungal and oomycete communities (Fig. 2, Supplementary Table 2). Beta-diversity, as determined using the Bray-Curtis dissimilarity metric, was not significantly impacted by drought, however there were significant differences in the composition of the bacterial, fungal and oomycete communities between UH and H soil (Fig. 3, Supplementary Table 3).

**Fig. 4:**
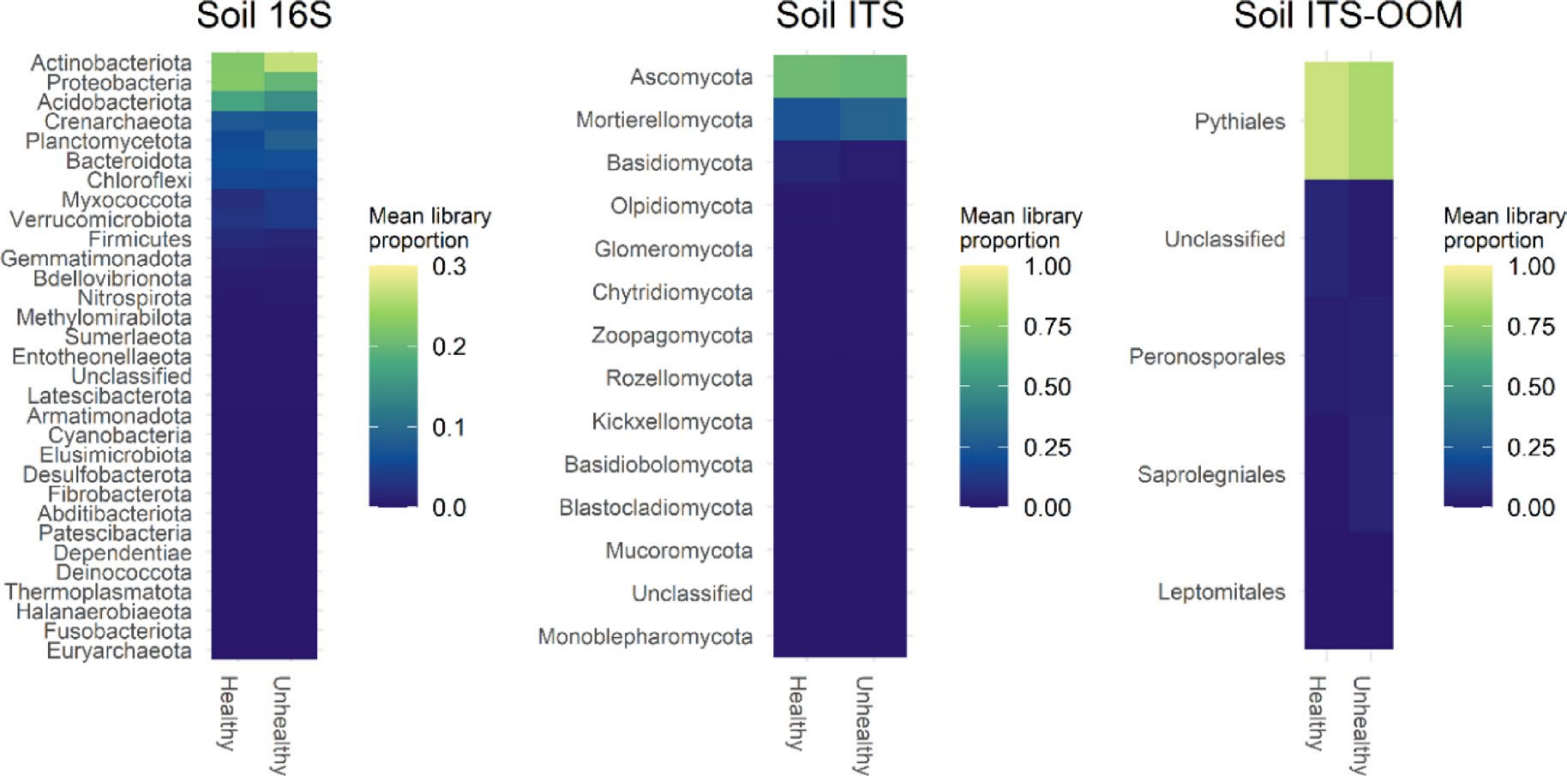
Mean relative taxonomic abundance within 16 S (Phylum), internal transcribed spacer (ITS) (Phylum) and ITS-oomycete (ITS-OOM) (Order) libraries for Healthy and Unhealthy soil samples. Taxonomic classification of all ASVs was done using Naïve Bayes Classification with the q2-classifier in QIIME2 and the SILVA (released 2022/09/12) and Unite (released 2021/10/05) reference sets respectively. All ASVs less than 100 bp as well as any 16 S ASVs that were classified as mitochondrial, or chloroplast sequences were removed.

**Fig. 5:**
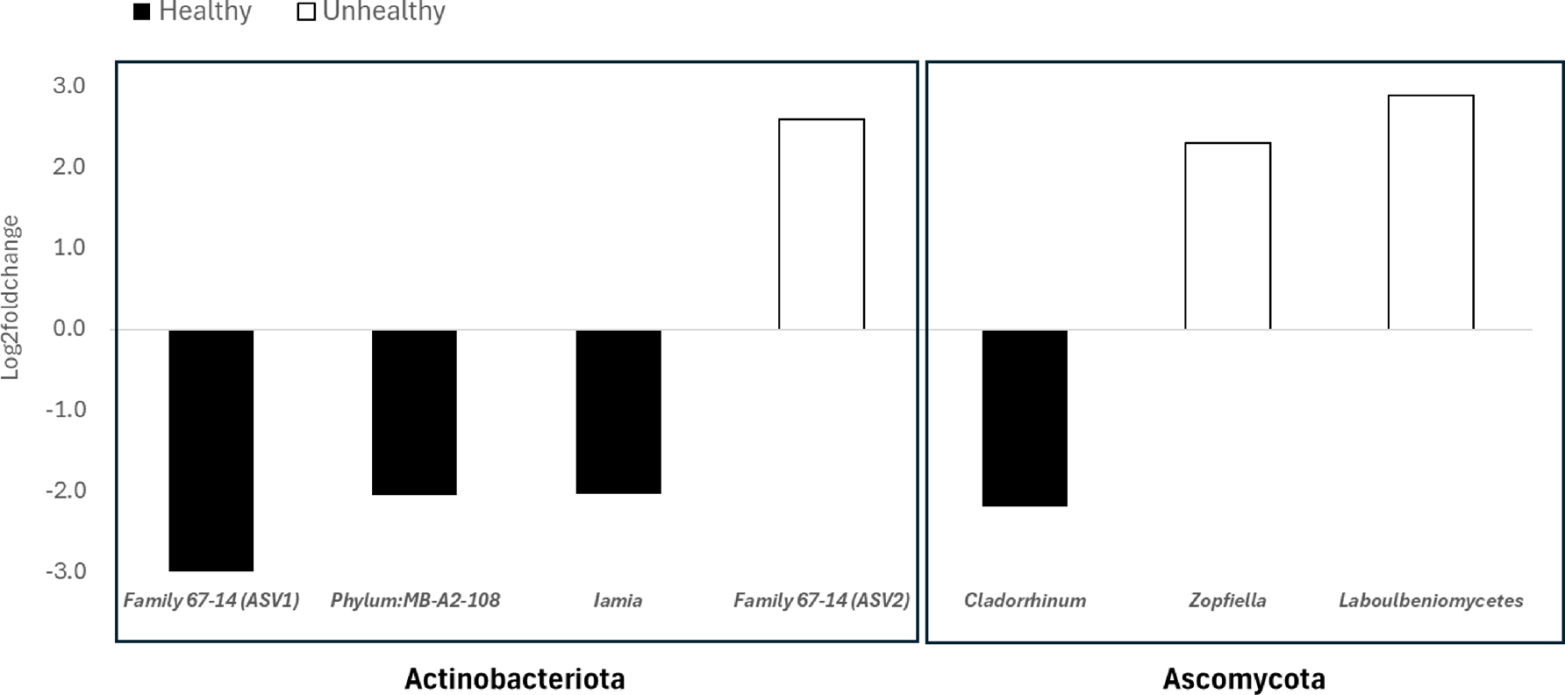
Differentially abundant bacterial (16 S) and fungal internal transcribed spacer (ITS) amplicon sequence variants (ASVs) identified by both ANCOMBC and Aldex2. For some groups, it was only possible to recognize class, order or Phylum: 67 − 14: Thermoleophilia, Solirubrobacterales, MB-A2-108: Actinobacteriota, Iamia: Acidimicrobiia, Microtrichales, Cladorrhinum: Sordariomycetes, Sordariales, Zopfiella: Sordariomycetes, Sordariales and Laboulbeniomycetes, Laboulbeniomycetes.

Further exploration of the compositional differences using both ALDex2 and ANCOM indicated there were no differences detected between H and UH at the phylum (16 S and ITS) or order (ITS-OOM) levels (Fig. 4, ANCOMBC BH *p* > 0.05), however multiple specific bacterial and fungal ASV sequences that were significantly more abundant in either H or UH soil samples were identified. All differentially abundant bacteria were Actinobacteriota, including two closely related ASV sequences of Solubacterales 64 − 17 that had 2 nucleotide differences from each other and were significantly different between H and UH soils. In the fungal community, one ASV classified as *Cladorrhinum* sp. was more abundant in H soil while two classified as *Zopfiella* sp. and *Laboulbeniomycetes* were more abundant in UH soil (Fig. 5). The analysis of soil microbiota did not identify any known pathogens of chickpea that were more abundant in the UH soil compared to the H soil, suggesting the symptoms were not related to the composition of the soil biota.

### Chickpea parameters (height, nodes, pods, and chlorophyll florescence)

**Fig. 6:**
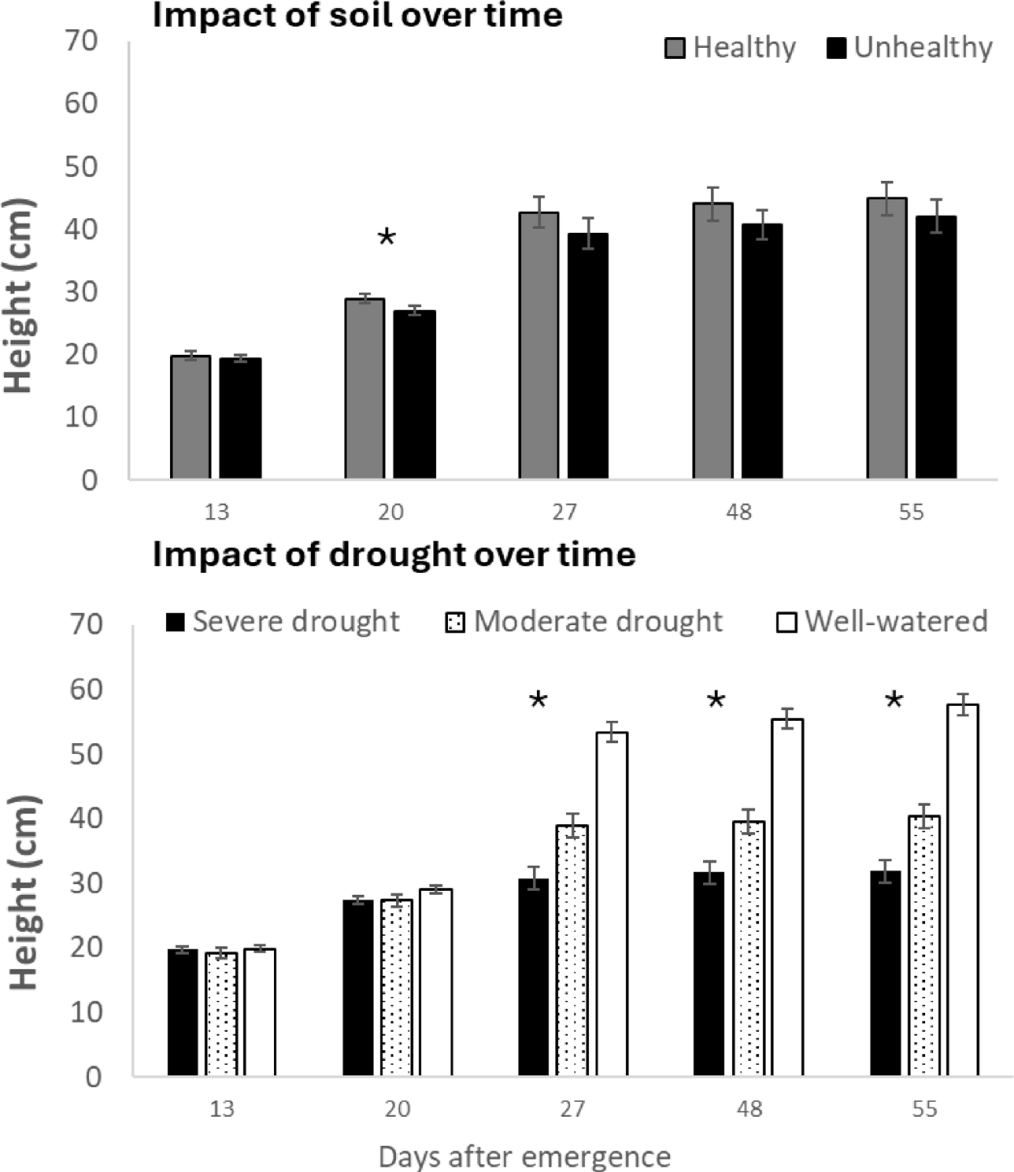
Height of chickpea plants growing in healthy (H) and unhealthy (UH) soil (average across all watering treatments, soil *p* < 0.01), and chickpea plants growing under severe drought, moderate drought and well watered conditions (averages for H and UH soil, water *p* < 0.01). Asterisk (*) indicate statistically significant differences between category when each date was compared separately (*p* < 0.05).

**Fig. 7:**
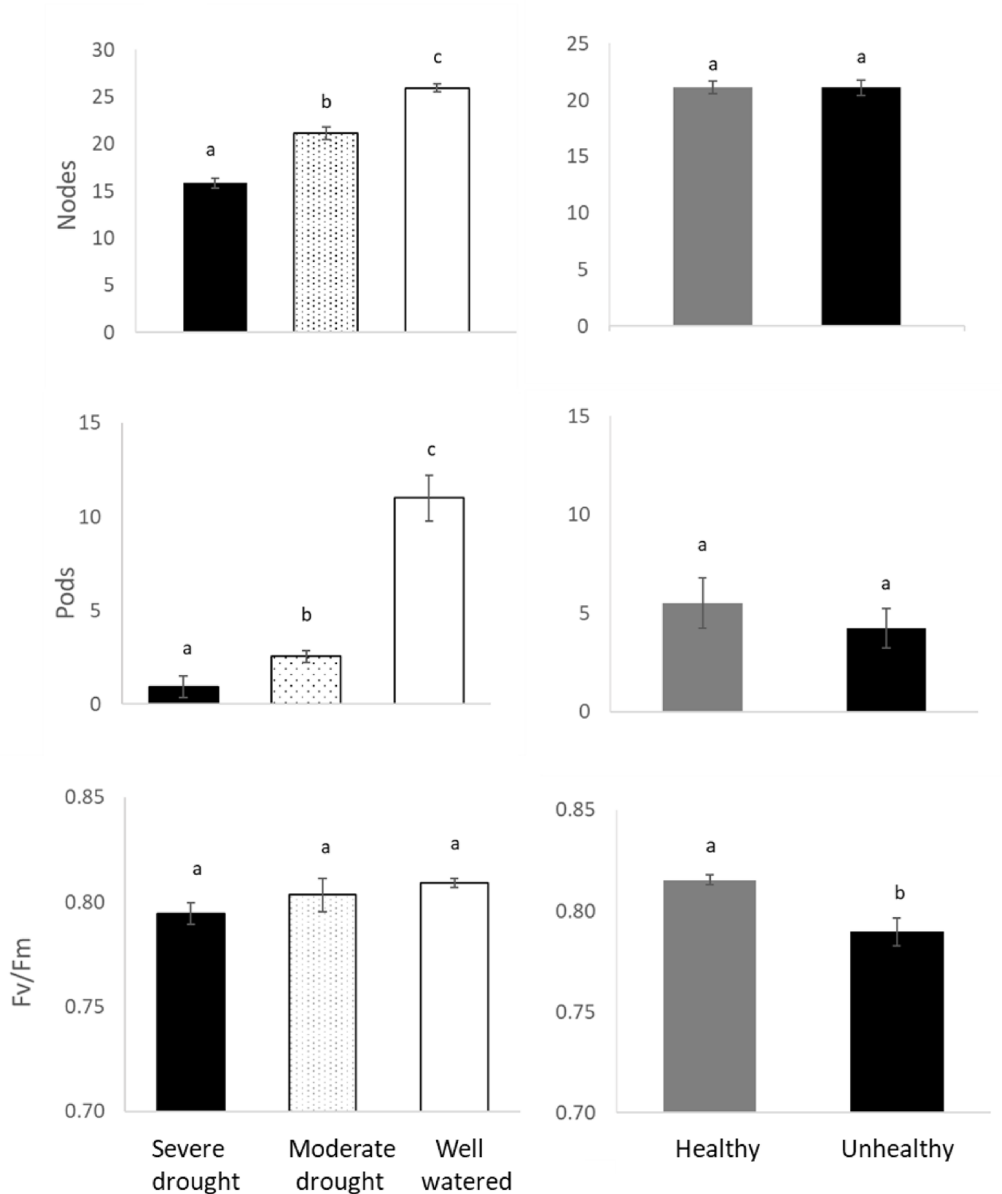
Nodes, number of pods and chlorophyll fluorescence ratio (Fv/Fm) for chickpea plants growing under severe drought, moderate drought and well-watered conditions (right panel) and healthy (H) and unhealthy (UH) soil (left panel). Within a panel, bars with the same letter are not statistically different (*p* < 0.05).

**Fig. 8:**
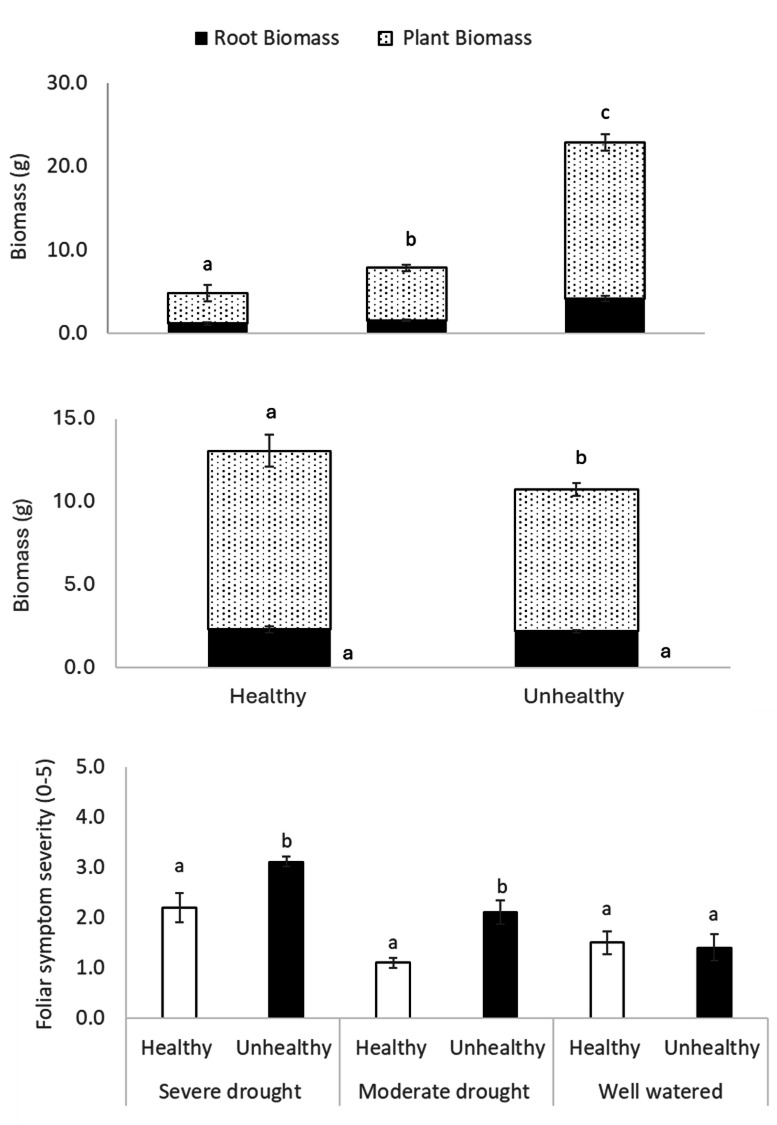
Chickpea above and belowground biomass for severe, moderate drought and well watered and H and UH soil. Foliar symptom severity consistent with drought for chickpea under severe or moderate drought or and well watered and in healthy and unhealthy soils. Within a panel, and parameter, bars with the same letter are not statistically different (*p* < 0.05).

The fact that chickpea plants were grown in pots in the greenhouse could have influenced the results by restricting the volume of soil available for the roots to colonize. Being in the greenhouse also meant that plants were not exposed to low temperatures that might occur in the field. All variables were negatively and significantly affected by drought in both H and UH soil. Plants in the UH soil had poorer performance compared to those in H soil, especially under drought conditions (Figs. 6, 7 and 8; supplementary Table 1). Plant height differed between soil type (soil *p* < 0.01, soil*time *p* = 0.30) and drought condition (drought *p* < 0.001, drought*time *p* < 0.001) (Fig. 6). The interactions between soil *drought (*p* = 0.27) as well as soil*drought*time (*p* = 0.66) were not significant. Chickpea height was significantly reduced by UH soil, relative to H soil, under moderate drought, but not under severe drought or well watered conditions (Supplementary Fig. 1).

The number of nodes and pods (assessed only once, near the end of the experiment) was drastically reduced under water stress (Fig. 7, *p* < 0.01 for both variables). There were fewer pods (-20%) on plants growing in UH soil; however, this difference was not statistically significant, probably due to the high variability between pots (*p* = 0.90). None of the interactions between soil and drought for nodes and pods was statistically significant (*p* = 0.27 and *p* = 0.35 for nodes and pods respectively). Chlorophyll fluorescence ratio Fv/Fm, differed significantly between H and UH soil (*p* < 0.01), but did not change with the drought treatment (*p* = 0.06) (Fig. 7).

Overall, plants in UH soil showed 20% less aboveground biomass than in H soil (*p* < 0.01), but no difference in belowground biomass detected (*p* = 0.79, Fig. 8). Under drought, plants in UH soils showed 50% less biomass but under the UH-well watered treatment this difference was reduced to 18% (Supplementary Fig. 2). However, these impacts were not statistically significant (*p* = 0.91).

### Chickpea health issue and drought status symptoms severity

The chickpea displayed foliar symptoms, including wilting and chlorosis. However, the symptoms did not match those of the chickpea emerging health issue and were more consistent with drought; wilting and discoloration occurring on older growth near the bottom of the plants, as opposed to near the tops. Leaf edge chlorosis or whitening was observed but also tended to be lower in the plants than would be expected for the chickpea emerging health issue (Supplementary Fig. 3). These symptoms were more severe for plants subjected to drought or growing in UH soil and their interaction (drought *p* = 0.01, soil *p* = 0.01, drought*soil *p* = 0.003; Supplementary Table 1). Under severe or moderate drought, but not in the absence of drought, chickpea grown in UH soil had more severe foliar symptoms related to more to general health-drought status (Fig. 8, *p* < 0.05). Chickpea root rot severity at the end of the study period was moderate, at 3.4 and 3.6 on a 1 to 7 scale for plants grown in H or UH soil and 3.8, 3.2 and 3.6 for severe drought, moderate drought and well watered, respectively. Severity of root rot was not impacted by drought or soil condition main effects (*p* = 0.14 and *p* = 0.57, respectively).

## Discussion

None of the chickpea plants in this study developed symptoms fully consistent with the emerging health issue of chickpea observed in southwestern Saskatchewan from 2019 to 2023. However, drought and its interaction with the soil (H or UH) impacted chickpea health, including foliar symptoms consistent with drought, such as wilting and discoloration low in the plant canopy (Supplementary Fig. 3). The negative impacts of drought on chickpea health are consistent with results from several studies^15–17^ in which drought reduced growth, photosynthetic performance, the number of pods and yield. It is surprising that drought did not reduce Fv/Fm. This parameter is typically responsive to drought, as damaged to photosystem II leads to lower Fv/Fm values. Potentially, the level of drought stress was not sufficient to induce significant, consistent damage to photosystem II. The fact that measurements of Fv/Fm were not taken from dead leaves, which would likely have measured zero, may have reduced the apparent impact of drought. Additional exploration of the drought tolerance of CDC Pearl, the chickpea variety used, relative to other varieties is merited; significant variety-to-variety variability exists in chickpea^18^. It is possible that CDC Pearl has a drought-resilient photosystem II.

Drought can also increase chickpea susceptibility to and/or yield losses from disease^19,20^. This is consistent with our finding that combined application of drought and UH soils led to worse chickpea performance. Conducting the experiments in pots may limited the impacts of UH soil by restricting root growth. In the field, chickpea roots would explore a deeper and wider soil area than that of the pots used. Under drought conditions in the field chickpea roots would penetrate very deeply into the soil in search of moisture. Feeding by ectoparasitic nematodes could reduce plant’s ability to do this. However, in pots, the differences between roots on which nematodes have fed and those on which they have not would be limited because of the pots.

Soil type (H or UH) influenced chickpea height over time, chlorophyll fluorescence and severity of symptoms consistent with drought. This is reasonable as it is well established that soil factors can impact plant performance^19^. For example, depletion of nutrients can be responsible for low performance and losses in chickpea yields^16,21,22^. In this experiment we observed twice as much K^+^ and SO_4_^−^ in the H soil compared to the UH soil. Both K^+^ and SO_4_^−^ play an important role increasing chickpea nodulation and yield^23,24^. Generally, K^+^ deficiency issues are flagged in soils with extractable exchangeable K^+^ levels less than about 200 mg K^+^/kg of soil and the UH soil had mean level of 174 mg K^+^/kg of soil. Potassium increases chickpea resistance to drought by maintaining higher water status under water stress via thermal protection of the synthetase activity^25^. This could help explain why the plants growing in H soil under severe or moderate drought, but not well watered conditions, performed better that those growing in the UH soil in terms of foliar symptoms of drought and general stress (Fig. 7). Concentrations of Mn^+^ and Mg^+^ were higher in H soil, which could contribute to improved chickpea resilience to drought in the H soil. Although magnesium deficiency is rare in chickpeas, this deficiency can reduce biomass and yield^26^. Manganese can enhance pod weight and protein content^27^. UH soil had higher levels of NO_3_^−^, PO_4_^−^ and Ca^+^, while these differences were statistically significant, overall, they were small in terms of likely biological importance. These differences could also be a result, rather than a cause, of the lower performance of the plants in UH soil; the plants in the UH may have taken up less of these nutrients due to reduced growth in the field. Although plant tissue nutrient concentrations were not measured in the current study and critical nutrient concentrations for chickpea cultivars are not available, a previous field study showed no systematic variation in nutrient concentrations between H and UH chickpea plants in a field survey^8^, although there was a significant relationship found between K concentration in tissue and severity of the symptoms observed.

Although texture significantly varied between H and UH soil, the differences were small and probably did not impact soil water retention. However, soil texture can impact nematode distribution. Nematodes abundance and density usually increase with sandier soils as it facilitates their active movement^28,29^, which may help explain why spiral nematodes were higher in UH soils compared to H ones. The difference in spiral nematode abundance between the H and UH soils contrasts with the finding of Marchesini et al., (2025) that *Helicotylenchus* (spiral) nematode density was not different between soils supporting chickpea with or without symptoms consistent with the emerging health issue.

Nematodes symptoms usually occur in patches across the fields, which is consistent with the observed damage linked to the chickpea emerging health issue. *Helicotylenchus*, which is among the most economically important ectoparasitic nematode genera^30^, has a broad host range, including chickpeas, common beans, peas, soybeans and lentils^31–34^. Nematode damage severity hinges on various factors, including nematode pathotype, crop species, nematode population densities, management practices, soil attributes, and climatic conditions^30^. Moreover, symptoms of nematode damage often mimic those caused by drought, nutrient deficiencies, and other stressors, complicating immediate determination of the cause(s) of symptoms^35^. Nematodes can reduce the ability of chickpea roots to absorb water and nutrients, rendering them even more vulnerable to drought conditions^36^. Root systems compromised by nematodes may not lead to obvious host damage if adequate moisture and nutrients are available; however, root damage exacerbates the effect of drought stress and nutrient deficiencies. Considering this, the elevated number of spiral nematodes found in the UH soil might play a role in plant performance and interactions between soil (H or UH) and drought. More studies are needed to confirm the role of nematodes, particularly the pin and spiral groups, in the occurrence and severity of the chickpea emerging health issue, and on the capacity of spiral nematodes to reproduce on chickpea. If the spiral nematodes observed in this study can reproduce on chickpea, this would increase the likelihood of them inducing symptoms. If nematodes do prove to be a significant health issue in chickpea, research on impacts of management tools on nematode populations and any symptoms they may produce in plants are also merited. Potentially management strategies in nematicides - such as carbofuran for soil fumigation^37^ - biocontrol, biofumigation, or fumigation with natural products, crop rotation, cover crops, or intercropping and/or alternative chickpea varieties or breeding for genetic resistance.

While there were significant differences in the composition of the bacterial, fungal and oomycetes communities, the results did not identify a microbial pathogen that was strongly associated with the UH soils and would have explained the symptoms observed. It is likely the differences in composition are related to the high number of nematodes in the UH soil compared to the H soil, however, was not possible to determine whether the microbiome or the nematodes are affecting the differences. In contrast to previous work investigating changes in the soil microbiome in response to drought^13^, we did not observe a significant effect on either microbiome diversity or composition in response to watering regime in either soil type. This may reflect the limitations of greenhouse experiments as the potential for soil microbial community turnover is limited compared to what would be expected in a field setting. However, it was interesting to note the number of Actinobacteriota that were more abundant in the H soils as members of this phylum, including those of the class *Thermoleophilia*, have been previously identified as potential plant growth-promoting bacteria^38^, and may be contributing to a more suppressive soil community. On the other hand, fungi from the class Laboulbeniomycetes are known to be obligate biotrophs of arthropods^39^. While Laboulbeniomycetes have not been specifically associated with nematodes, their increased abundance is interesting in the context of the higher numbers of spiral nematodes found in the UH soil (Fig. 3). A study with cover plants and cucumber showed that the presence of beneficial fungal in the rhizomicrobiome as *Cladorrhinum a*nd *Zopfiella* suppress fusarium wilt and root-knot nematodes, highlighting the potential relationship *Zopfiella* (which was more abundant in H soils in this study) and nematodes^40^.

In conclusion, our results suggest that spiral nematodes, associated differences in soil microorganisms and/or lower levels of K^+^ and SO_4_ in the UH soil reduced chickpea capacity to cope with drought. Some or all of these factors may contribute to the emerging health issue in chickpea. However, other factor(s), such as the absence of the root-confining impacts of being grown in pots, are also needed for the development of the distinctive symptoms of the chickpea emerging health issue concentrated on new plant growth.

## Materials and methods

### Soil collection

Soil was collected from South East Research Farm (SERF) in Redvers, Saskatchewan, Canada (49.54 N, 101.66 W) in September 2023, immediately after chickpea harvest. The plots from which the soil (both unhealthy (UH) and healthy (H)) was collected were planted to CDC Pearl (kabuli chickpea cultivar) in a regional variety trial, comparing different varieties of chickpeas; no other treatments were applied to any plots. Thus, both H and UH soils were collected from the same treatment, but different replicates. The soils in this area are black chernozemic, loamy texture and moderately stony^41^. The pH ranged from 6.8 to 7.5 and there was gentle sloping topography in the area. The soil collection took place in UH plots where symptoms consistent with the chickpea emerging health issue were present or in H plots where these symptoms were absent.

The symptoms were chlorosis of leaf outer edges, necrosis of leaf tips, generally concentrated near the tops of the plants (Fig. 1B), as opposed to lower down, as would be expected from other stressors, such as drought. Each plot was 5 m^2^ (3.6 × 1.4 m) and the distance between H and UH collection areas was approximately 25 m, with minimal difference in elevation or topography (Fig. 1A). The area within the plots from which soil was collected were roughly 1–2 m^2^. No difference in the level of drought is suspected between the two sites. Precipitation in 2023 in Redvers was below the long-term average (Supplementary Table 4). Because of this, and the occurrence of early-season drought in other sites and years where the chickpea emerging health issue has appeared, we hypothesize that drought and soil interact to contribute to the symptoms. Soil was collected from the top 30 cm and placed in separated containers to avoid cross contamination. The soil was kept in a cold dark room (4 °C) until it was homogenized and subsequently placed in pots for the greenhouse experiment.

### Greenhouse experiments

Two experiments were performed at the AAFC Swift Current Research and Development Centre greenhouse facilities, in Swift Current, Saskatchewan, Canada. The experimental design was a two-factor factorial arranged as a randomized complete block with five blocks (pots), and the trial was repeated. Thus, there were five pots for each soil and water combination in each experiment. The first factor was soil type with two conditions of previous chickpea growth: healthy (H) and unhealthy (UH). The second factor was water level: well-watered (W), moderate drought (M), and severe drought (D). The first experimental run took place from September 29 to November 30, 2023, and the second from November 20, 2023, to January 17, 2024. When each trial was terminated, the most mature plants have green pods, meaning they have reached or passed the development stages at which the chickpea emerging health issue symptoms typically develop.

Soil was sieved using a 2 mm sieve and the pots were watered to field capacity. Three seeds of CDC Pearl chickpea were planted per pot and 24.4 mg of granular inoculant (TagTeam BioniQ-Canada, Novozymes BioAg, 3935 Thatcher Avenue, Saskatoon, SK Canada S7R 1A3) was added to each 4 L pot. After 13 or 10 days (first and second experiments, respectively), plants were randomly thinned, leaving one seedling per pot. All pots were well-watered for the first 14 or 16 days to ensure emergence. Subsequently, drought treatments – during which plants were watered three days a week, was initiated. Soil field capacity was 720 ml per pot, and each pot contained approximately 3.0 kg of dry soil. Water levels were set at 13% of the pots field capacity (90 ml of water per pot at the beginning of the experiment) for the severe drought treatment, 25% (180 ml) for the moderate drought and 40% (240 ml of water per pot) for the well-watered treatment, administered on each application, respectively. However, the watering volume changed as the plants developed, from 90, 150 and 240 to 120, 180 and 350 ml per treatment. Plants were kept at 22–23 °C and 18–20 °C day and night temperatures respectively, with a photoperiod of 12–14 h (23.900 lx on average).

### Soil nutrients, EC and pH

Three soil samples (100 g each) per soil condition (H or UH) were taken from the bulk soil used for potting (the soil was kept cold prior to analysis) and analyzed for nutrients at the end of the experiment. The SO_4_-S, NO_3_-N and PO_4_-P were measured using a SEAL AutoAnalyzer 3 Continuous Segmented Flow Analyzer (SEAL Analytical, 6501 West Donges Bay Road, Mequon, Wisconsin, US) following the procedure established by Hamm et al., (1970)^42,43^, Harm et al. (1973)^53^ and Gentry and Willis (1988)^44^. The K^+^ analysis were performed by Atomic Absorption Spectroscopy (ThermoFisher iCE3300, Thermo Fisher Scientific, 168 3rd Ave, Waltham, US) according to the standard equipment operating setup^42^. Ca^+^, Mg^+^ and Na^+^ were extracted in 1 N ammonium acetate and analyzed using a Thermo Scientific iCAP6300 Duo Inductively Couple (ICP) Plasma Spectrometer^45^. Micronutrients (Cu^+^, Fe^+^, Zn^+^ and Mn^+^) were extracted by atomic absorption spectroscopy^46^. Total and organic carbon were measured using an Elementar vario MICRO cube elemental analyzer^47^. Electrical conductivity on saturated paste extract and pH on saturated paste was measured following McKeague (1978)^46^. Texture was determined by the particle size determination method^48^.

### Nematodes

Three soil samples for each of H or UH condition from the bulk soil used for potting (i.e. before the experiments) were sent to Dr. Mario Tenuta’s laboratory at University of Manitoba for nematodes analysis. Nematodes were extracted from 100 g of soil using the sieving and sugar centrifugation method^49^ and counted. The first 100 nematodes were identified to genus using morphological characteristics and standard taxonomic keys^50^. Soil moisture content was determined for reporting nematode abundance on a dry soil basis. Nematodes were not quantified in the soil collected from the pots after the experiments.

### Bacterial, fungal and oomycete community composition

For each sample, collected after the experiment was complete, total genomic DNA was extracted from 250 mg of soil using the Qiagen Power Soil Kit as per the manufacturer’s instructions (Qiagen, Germany). Thirty samples were used in total, 15 from each H-UH condition. Of these 15 samples, five came from well-watered, five from moderate drought and five from severe drought. The samples came from the second-last experiment only. DNA samples were diluted 1/10 in molecular biology grade water (ThermoFisher Scientific, MA, USA) and amplified with 16 S (V4), ITS and oomycete-specific ITS primers (Table 2). PCR amplicons were barcoded with Illumina Nextera XT v2 indexes and sequenced using Illumina V2 250PE chemistry (Illumina, CA, USA). Demultiplexed sequencing reads were trimmed with Cutadapt (v.2.8)^51^ to remove amplification primer sequences as well as sequence with an average quality score < Q30. R1 and R2 reads were merged using FLASH2 (v.2.2)^52^ and amplicon sequence variants (ASVs) for 16 S, ITS and oomycete (OOM) targets were identified using the q2-DADA2^53^ plugin for QIIME2 (v.2021.2) ^52^. After quality control and data processing, library sizes range from 5,033 − 23,974 for 16 S, 5,723 − 10,221 for ITS, and 1,071 − 9,458 for ITS-OOM. Taxonomic classification of all ASVs was done using Naïve Bayes Classification with the q2-classifier in QIIME2 and the SILVA (released 2022/09/12)^54^ and Unite (released 2021/10/05)^55^ reference sets respectively. All ASVs less than 100 bp as well as any 16 S ASVs that were classified as mitochondrial, or chloroplast sequences were removed.

**Table 2:** Group, sequence and cycles characteristics for bacterial (16 S), fungal internal transcribed spacer (ITS) and oomycetes (ITS) amplification.

Group		Sequence	Cycle	References
16 S (V4)	515 F 806R	GTGYCAGCMGCCGCGGTAA GACTACHVGGGTWTCTAAT	30 cycles: 95 °C 30s, 55 °C 30s, 72 °C 30s	^63^
ITS	ITS1f ITS2	CTTGGTCATTTAGAGGAAGTAA GCTGCGTTCTTCATCGATGC	30 cycles: 95 °C 30s, 55 °C 30s, 72 °C 30s	^64^
ITS-oomycetes	ITS6 ITS7ae	GAAGGTGAAGTCGTAACAAGG WGYGKTCTTCATCGATGTGC	30 cycles: 95 °C 30s, 55 °C 30s, 72 °C 30s	^65^

### Chickpea plant height, nodes, chlorophyll fluorescence, foliar and root symptoms

For each plant, the height, number of nodes and pods, chlorophyll fluorescence (Fv/Fm), foliar symptoms, root rot severity and the above and below ground biomass were measured. Natural plant canopy height (not stretched out) was measured with a measuring tape from the bottom of the plant up to the top. Chlorophyll fluorescence was measured using an optic fluorometer (OS30p+, Opti-Sciences, Inc.) when the plants had reached, or were nearing, early podding, zero to two days before the experiment was terminated and destructive sampling for biomass data was conducted. Leaves were dark adapted prior to measurement. Data was not collected from completely dead leaves. Height measurements were performed weekly; the rest of the variables were measured at the end of the experiment. After each experiment, plants were carefully harvested, and roots were gently washed to remove soil. Root rot was rated on a 1–7 scale^56^. Foliar symptoms were also rated repeatedly, but only data from the rating at the end of the experiment is presented. Foliar symptoms consistent with drought (yellowing near the base of the plants as opposed to the apex, decreased distance between nodes) were rated on a 0–5 scale, where 0 indicates the absence of symptoms. 1 indicates < 10% plant area unhealthy (wilted, discolored, yellow, chlorotic or necrotic); 2 indicates < 20% of plant area unhealthy; 3 indicates 30–60% lant area unhealthy; 4 indicates 60–80% lant unhealthy; and 5 indicates the plant is dead or almost dead.

### Statistical analysis

All analysis were performed using R studio^57^. Both experimental runs were combined so the number of replicates per treatment (combination of soil and watering regime) was 10. Experiment was not considered a fixed or random effect in the model; in preliminary analysis, each experiment was analysed separately and the results compared to assess the repeatability. For height, the data was analysed using a mixed linear model with water levels, soil and time as fixed effects and block (replicate) as a random effect (using the default unstructured covariance matrix). The interaction between water level, soil type and time and the three-way interaction were included for height, but not the other variables. For the response variables of nodes, pods, Fv/Fm, belowground biomass, foliar symptom and root rot severity we used the non-parametric Aligned Rank Transform test (ART) from the AR-tool package in R and the post-hoc pairwise associated tests. Although Fv/Fm and belowground biomass are continuous variables, they did not meet the assumption of normally distributed residuals or variance homoscedasticity. Thus, we used a non-parametric test that permits analysing multifactorial designs and testing interactions between factors for variables that don’t meet the parametric tests assumptions^58,59^. Soil texture, soil nutrients and aboveground biomass were analysed using ANOVA test. Tukey test for post-hoc comparisons were applied. Nematodes abundance between H and UH soil was compared using the Kruskal-Wallis non-parametric test^58^. Nematodes density was quantified only in field soil collected prior to the experiment, not in soil collected from the pots after the completion of the greenhouse experiments.

To examine differences in alpha and beta diversity of soil microbial communities, libraries were rarefied to the same number of reads: 5000 reads/sample for 16 S and ITS, and 1000 reads/sample for ITS-OOM (Supplementary Fig. 4). Bray-Curtis dissimilarity and Shannon diversity metrics were calculated using the ‘phyloseq’^60^ and ‘vegan’^61^ packages in R. Statistical significance for the Shannon index was evaluated with the Kruskal-Wallis test. The homogeneity of group Bray-Curtis dispersion values were confirmed using the *betadisper* function from the ‘vegan’ package for 16S, ITS and ITS-OOM targets (p > 0.05), and permutational multivariate analysis of variance (PERMANOVA) was used to analyze Bray-Curtis dissimilarity. Principal coordinate analysis (PCoA) was used to visualize the Bray-Curtis dissimilarity data (Supplementary Fig. 4). Differential abundance analysis of ASVs was conducted using centred log-ratio transformed abundance data with both the ‘ALDEx2’ and ANCOMBC^62^ packages in R, with only ASVs identified by both programs labelled as differentially abundant between H and UH soils (Benjamini-Hochberg corrected p < 0.05).

## Supplementary Information

Below is the link to the electronic supplementary material.

Supplementary Material 1

## Data Availability

The datasets generated and/or analyzed during the current study are available in the NCBI SRA repository under project number PRJNA1134170 link: https://www.ncbi.nlm.nih.gov/bioproject/PRJNA1134170 The other datasets generated during and/or analyzed during the current study are available from the corresponding author on reasonable request.
