# Supplementary material for: Spiral nematodes, soil microbiome and micronutrients increase chickpea drought susceptibility but do not induce symptoms of the emerging health issue
Source: Sci Rep. 2025 Nov 13;15:39823. doi: 10.1038/s41598-025-23475-0 (PMC12615619; doi:10.1038/s41598-025-23475-0)
Supplement: Supplementary file 1 — Supplementary Material 1 [file 41598_2025_23475_MOESM1_ESM.docx]

**Supplementary material**

Supplementary Table 1: ANOVA, AR-Tool and non-parametric results for height, nodes, pods, Fv/Fm, root biomass, nutrients and nematodes

| **Height** | **Num F** | **Den F** | **F-value** | **p-value** |
| --- | --- | --- | --- | --- |
| Water | 2 | 282 | 78.87 | <.0001 |
| Soil | 1 | 282 | 6.81 | 0.0095 |
| Time | 1 | 282 | 316.29 | <.0001 |
| Water*Time | 2 | 282 | 38.00 | <.0001 |
| Soil*Time | 1 | 282 | 1.06 | 0.3042 |
| Water*Soil | 2 | 282 | 1.31 | 0.271 |
| Water*Soil*Time | 2 | 282 | 0.41 | 0.6633 |

| **Nodes** | **F** | **DF** | **Df.res** | **Pr(>F)** |
| --- | --- | --- | --- | --- |
| Water | 72.48504 | 2 | 166.26 | <0.0002 |
| Soil | 0.010602 | 1 | 166.22 | 0.91811 |
| Water*Soil | 1.300171 | 2 | 166.22 | 0.27524 |
|  |  |  |  |  |
| **Fv/Fm** |  |  |  |  |
| Water | 3.00859 | 2 | 46.536 | 0.059049 |
| Soil | 18.16482 | 1 | 46.284 | 0.00009 |
| Water* Soil | 0.44476 | 2 | 46.261 | 0.64369 |
|  |  |  |  |  |
| **Biomass** |  |  |  |  |
| Water | 28.81931 | 2 | 49.135 | 0.0000001 |
| Soil | 0.066625 | 1 | 49.138 | 0.79739 |
| Water*Soil | 0.084241 | 2 | 49.135 | 0.91934 |
|  |  |  |  |  |
| **Foliar symptom severity** | | | |  |
| Water | 20.7938 | 2 | 49.042 | 0.000002 |
| Soil | 10.9692 | 1 | 49.042 | 0.0017445 |
| Water*Soil | 6.4334 | 2 | 49.038 | 0.0033028 |

| **Nutrients** |  |  |  |  |  |
| --- | --- | --- | --- | --- | --- |
| **Soil SO_4-_** | **df** | **Sum** | **Sq Mean** | **Sq F** | **Pr>F** |
| Soil | 1 | 33.61 | 33.61 | 34.77 | 0.00414 |
| Residuals | 4 | 3.87 | 0.97 |  |  |
|  |  |  |  |  |  |
| **Soil Cu+** |  |  |  |  |  |
| Soil | 1 | 1.4113 | 1.4113 | 6.002 | 0.0704 |
| Residuals | 4 | 0.9405 | 0.2351 |  |  |
|  |  |  |  |  |  |
| **Soil Fe+** |  |  |  |  |  |
| Soil | 1 | 11.207 | 11.207 | 5.156 | 0.0857 |
| Residuals | 4 | 8.693 | 2.173 |  |  |
|  |  |  |  |  |  |
| **Soil Mn+** |  |  |  |  |  |
| Soil | 1 | 90.48 | 90.48 | 28.8 | 0.00582 |
| Residuals | 4 | 12.57 | 3.14 |  |  |
|  |  |  |  |  |  |
| **Soil Zn+** |  |  |  |  |  |
| Soil | 1 | 0.8817 | 0.8817 | 5.813 | 0.0735 |
| Residuals | 4 | 0.6067 | 0.1517 |  |  |
|  |  |  |  |  |  |
| **Soil K+** |  |  |  |  |  |
| Soil | 1 | 40623 | 40623 | 2426 | 1.02E-06 |
| Residuals | 4 | 67 | 17 |  |  |
|  |  |  |  |  |  |
| **Soil NO_3_-** |  |  |  |  |  |
| Soil | 1 | 30.827 | 30.827 | 28.32 | 0.006 |
| Residuals | 4 | 4.353 | 1.088 |  |  |
|  |  |  |  |  |  |
| **Soil PO_4_-** |  |  |  |  |  |
| Soil | 1 | 54 | 54 | 36.99 | 0.00369 |
| Residuals | 4 | 5.84 | 1.46 |  |  |
|  |  |  |  |  |  |
| **Soil Ca+** |  |  |  |  |  |
| Soil | 1 | 6062160 | 6062160 | 810.7 | 9.05E-06 |
| Residuals | 4 | 29910 | 7478 |  |  |
|  |  |  |  |  |  |
| **Soil Mg+** |  |  |  |  |  |
| Soil | 1 | 129772 | 129772 | 922.4 | 7.00E-06 |
| Residuals | 4 | 563 | 141 |  |  |
|  |  |  |  |  |  |
| **Soil Na+** |  |  |  |  |  |
| Soil | 1 | 45.93 | 45.93 | 44.09 | 0.00267 |
| Residuals | 4 | 4.17 | 1.04 |  |  |
|  |  |  |  |  |  |
| **Soil Total-C** |  |  |  |  |  |
| Soil | 1 | 0.00167 | 0.001667 | 0.143 | 0.725 |
| Residuals | 4 | 0.04667 | 0.011667 |  |  |
|  |  |  |  |  |  |
| **Soil Org-C** |  |  |  |  |  |
| Soil | 1 | 0.4267 | 0.4267 | 32 | 0.00481 |
| Residuals | 4 | 0.0533 | 0.0133 |  |  |
|  |  |  |  |  |  |
| **Soil EC** |  |  |  |  |  |
| Soil | 1 | 0.04167 | 0.04167 | 25 | 0.00749 |
| Residuals | 4 | 0.00667 | 0.00167 |  |  |
|  |  |  |  |  |  |
| **Soil pH** |  |  |  |  |  |
| Soil | 1 | 0.00667 | 0.00666 | 4 | 0.116 |
| Residuals | 4 | 0.00667 | 0.00167 |  |  |
|  |  |  |  |  |  |

| **Texture** |  |  |  |  |  |
| --- | --- | --- | --- | --- | --- |
| **% Sand** | |  |  |  |  |
| Kruskal-Wallis chi-squared = 3.9706, df = 1, p-value = 0.0463 | | | | | |
| **% Clay** | |  |  |  |  |
| Kruskal-Wallis chi-squared = 0.047619, df = 1, p-value = 0.8273 | | | | | |
| **% Silt** |  |  |  |  |  |
| Kruskal-Wallis chi-squared = 3.8571, df = 1, p-value = 0.04953 | | | | | |

| Abbreviations:  Soil Total-C Soil Total Carbon;  Soil Org-C Soil Organic Carbon;  Soil EC Soil electrical conductivity  **Nematodes:**  **Paratylenchus** |  |  |
| --- | --- | --- |
| data: Paratylenchus by Soil health status | | |
| K- chi-squared = 0.78431, df = 1, p-value = 0.3758 | | |
|  | | |
| **Helicotylenchus** |  |  |
| data: Helicotylenchus by Soil health status | | |
| K-W chi-squared = 4.3548, df = 1, p-value = 0.0369 | | |

Supplementary Table 2: Kruskal Wallis analysis of Shannon diversity metric for bacterial (16S), fungal internal transcribed spacer (ITS) and oomycete (ITS-OOM) soil communities between healthy (H) and unhealthy (UH) soils or between severe drought (D), moderate drought (M) and well-watered (W) treatments.

|  | 16S | | ITS | | ITS-OOM | |
| --- | --- | --- | --- | --- | --- | --- |
|  | χ^2^ | p-value | χ^2^ | p-value | χ^2^ | p-value |
| Soil (H, UH) | 0.17 | 0.896 | 0.96 | 0.328 | 0.03 | 0.854 |
| Drought (D, M, W) | 1.01 | 0.604 | 3.80 | 0.150 | 1.02 | 0.601 |
| Soil*Drought | 5.16 | 0.396 | 6.612 | 0.251 | 4.156 | 0.527 |

Supplementary Table 3: PERMANOVA analysis of the impacts soil, drought and the interaction between soil and drought on the Bray Curtis dissimilarity metric for bacterial (16S), fungal internal transcribed spacer (ITS) and oomycete (ITS-OOM) soil communities. Soil was either healthy (H) or unhealthy (UH). There were three levels of drought: severe drought (D), moderate drought (M) and well-watered (W). Statistically significant results are indicated in bold.

| Treatments | 16S | | | ITS | | | ITS-OOM | | |
| --- | --- | --- | --- | --- | --- | --- | --- | --- | --- |
|  | R^2^ | F | p-value | R^2^ | F | p-value | R^2^ | F | p-value |
| Soil (H, UH) | **0.09** | **2.79** | **0.01** | **0.11** | **3.02** | **0.01** | **0.06** | **1.60** | **0.04** |
| Drought (D, M, W) | 0.07 | 1.03 | 0.356 | 0.07 | 0.90 | 0.695 | 0.07 | 0.90 | 0.64 |
| Soil*Drought | 0.07 | 1.09 | 0.245 | 0.06 | 0.86 | 0.816 | 0.08 | 1.11 | 0.24 |


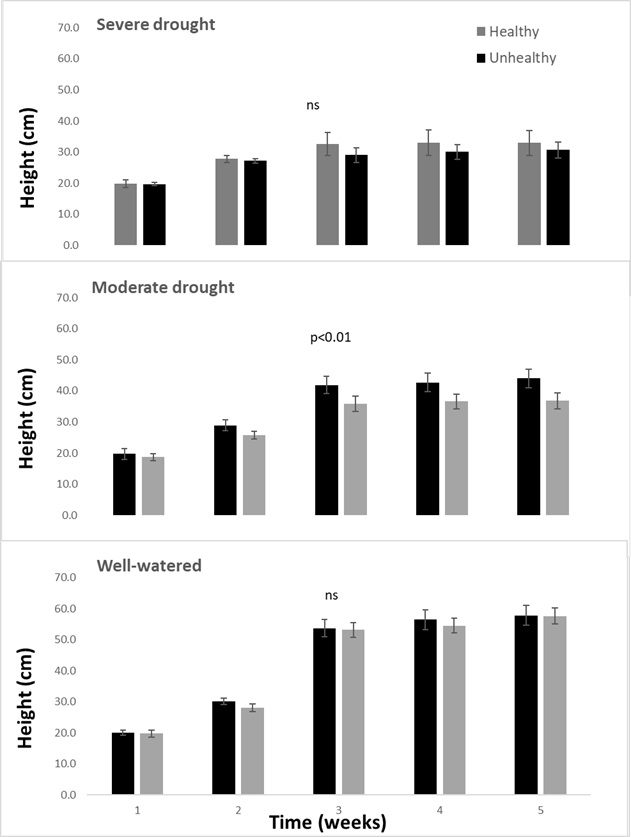


Supplementary Fig 1: Height of chickpea plants growing in healthy (H) or unhealthy (UH) soil under three different drought treatments (severe drought, moderate drought and well-watered).


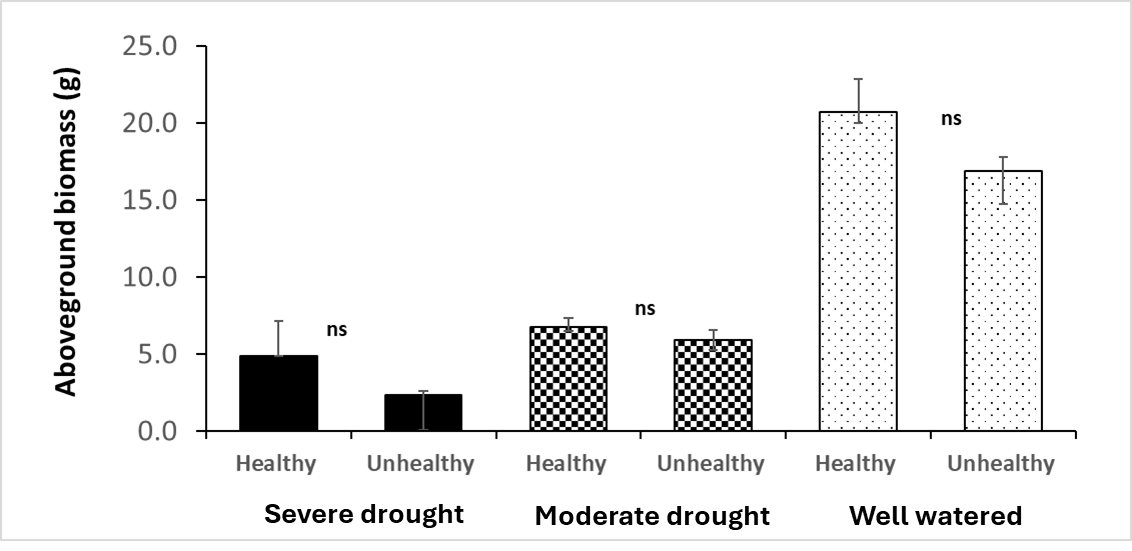


Supplementary Fig 2: Aboveground biomass (g) for chickpea’s plants growing in healthy (H)-unhealthy (UH) soils and under severe and moderate drought (D and M, respectively) and well-watered (W) treatments, respectively. Comparison between groups (DH-DUH, MH- MUH and WH-WUH). ns means not statistically significant.


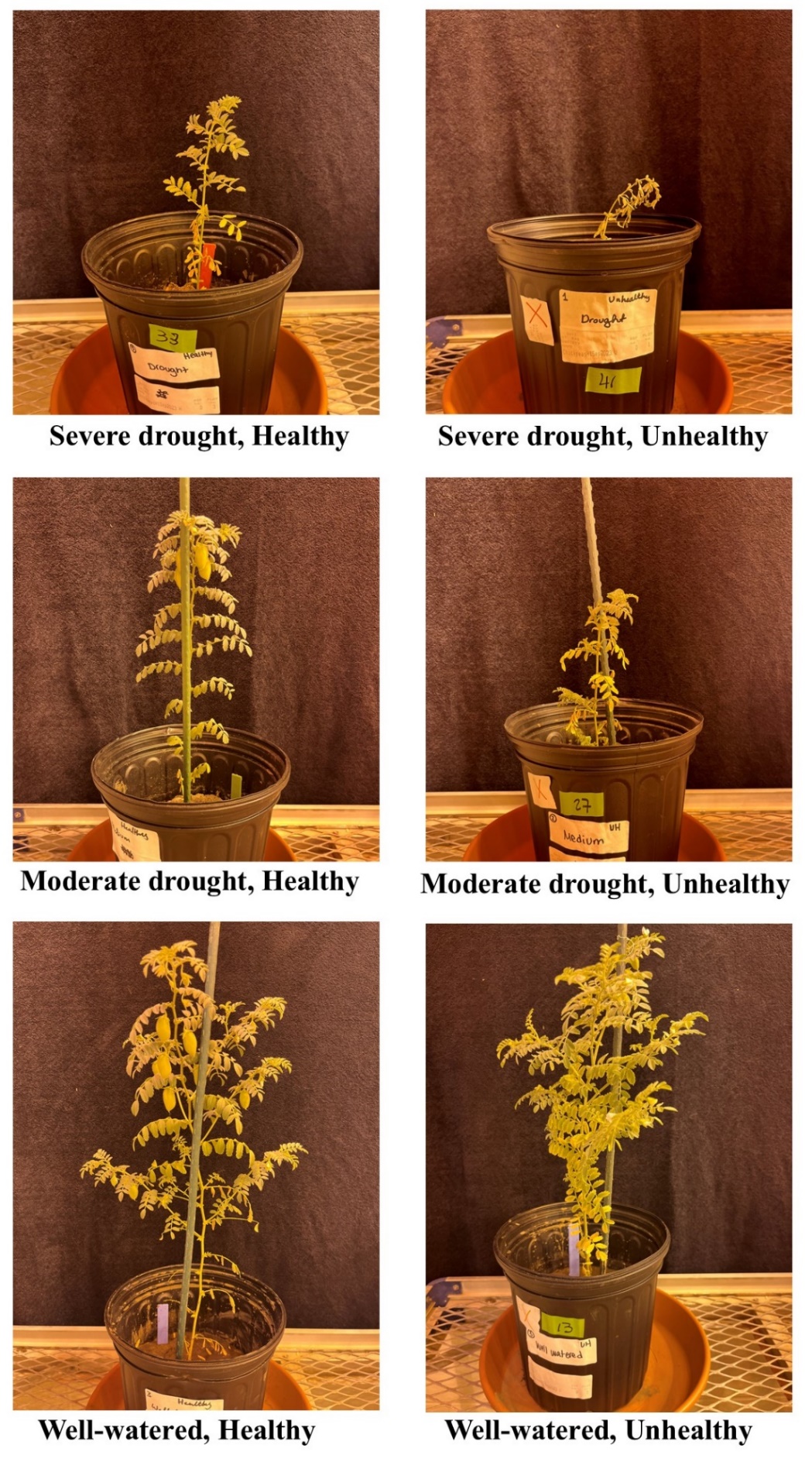


Supplementary Fig 3: Examples of foliar symptoms from plants under severe and moderate drought, or well-watered conditions, grown in healthy or unhealthy soil.

Supplementary Table 4. Comparison between monthly historical precipitation (mm) for Redvers (1981-2021) and Estevan (1994-2020) and precipitation for Redvers in 2023.

| **Precipitation (mm)** | **Jan** | **Feb** | **Mar** | **Apr** | **May** | **Jun** | **Jul** | **Aug** | **Sep** | **Oct** | **Nov** | **Dec** | **Year** |
| --- | --- | --- | --- | --- | --- | --- | --- | --- | --- | --- | --- | --- | --- |
| **Redvers 2023** | 0.5 | 1.5 | 8.9 | 33.0 | 0.0 | 84.1 | 10.8 | 37.6 | 35.2 | 32.2 | 4.2 | 7.2 | **255.2** |
| **Redvers 1983-2010** | 20.0 | 11.5 | 19.2 | 22.8 | 60.0 | 95.2 | 65.5 | 46.6 | 32.7 | 27.0 | 20.0 | 23.3 | **443.8** |
| **Estevan 1994-2020** | 16.7 | 14.9 | 18.9 | 27.3 | 65.7 | 84.7 | 66.2 | 47.4 | 45.5 | 29.2 | 19.9 | 18.4 | **454.8** |


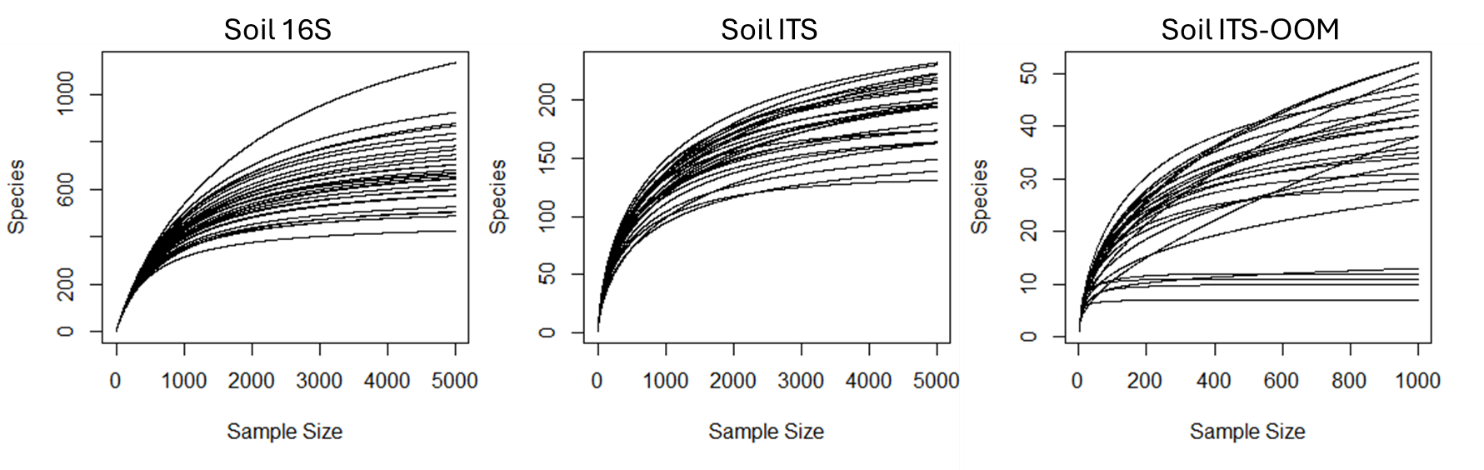
 Supplementary Fig 4: Rarefaction curves were calculated for 16S, internal transcribed spacer (ITS) and ITS-oomycete (ITS-OOM) libraries based on the number for reads for the smallest library, 5000 reads/sample for 16S and ITS, and 1000 reads/sample for ITS-OOM.
